# Clinical Care and Rehabilitation in Neuromuscular Disorders—Why It Highly Matters

**DOI:** 10.3390/jcm15093523

**Published:** 2026-05-05

**Authors:** Hans-Jürgen Gdynia

**Affiliations:** Department of Neurology, m&i-Fachklinik Enzensberg, 87629 Hopfen am See, Germany; hans-juergen.gdynia@fachklinik-enzensberg.de; Tel.: +49-8362123148; Fax: +49-8362123137

## 1. Neuromuscular Disorders—The Neglected Stepchildren of Neurology?

Neuromuscular disorders represent a diverse group of diseases mainly characterized by progressive or non-progressive muscle weakness. They can be categorized as diseases involving the peripheral nervous system, neuromuscular transmission, or muscles. Many of these conditions are very rare; consequently, there are scarce data on epidemiological frequencies and in particular on incidence. A recent study reported the annual incidence rates of eight disorders, ranging from 0.3 in 100,000 people for progressive (spinal) muscular atrophy and facioscapulohumeral muscular atrophy to 1 in 100,000 for Charcot–Marie–Tooth disease type 1 and myotonic dystrophy type 1 [1]. These diseases have highly diverse causes, including genetic defects that lead to conditions such as limb girdle muscular dystrophies [2] and spinal muscular atrophies [3], while many forms of inflammatory myopathies are autoimmune in origin [4]. Additionally, many peripheral neuropathies are acquired [5]. Many neuromuscular diseases have a progressive nature and a high degree of severity, frequently resulting in permanent disability or even death. A notable example of this is amyotrophic lateral sclerosis (ALS) [6,7]. In many cases, a significant delay may occur between the onset of symptoms and the establishment of an accurate diagnosis. A study of Fukutin-related protein-associated muscular dystrophy revealed a median diagnostic delay of 6.5 years in the cohort under investigation, with an interquartile range of 1–16 years [8]. This situation remains very challenging for those affected. Historically, neuromuscular disorders played a minor role in the field of neurology because only few diseases had therapeutic options available. For this reason, they were long regarded as the neglected stepchildren of neurology, receiving very little attention. Only a limited number of academic clinics and outpatient departments with designated consultation hours were specialized in the management of these conditions.

But circumstances have changed dramatically in recent decades.

## 2. Neuromuscular Disorders Are Becoming Increasingly Treatable!

Advances in modern medicine and the neurosciences, particularly neurogenetics, have led to a steadily growing understanding of the pathophysiology of these diseases. This has resulted in improved diagnostic and therapeutic options, even for diseases with a previously poor prognosis. Particularly noteworthy are the therapeutic advances in the treatment of genetically determined neuromuscular disorders like spinal muscular atrophy, which cannot yet be cured but can be treated causally using gene therapy approaches [3,9]. Furthermore, significant scientific progress has been made in exon-skipping for Duchenne muscular dystrophy (DMD) [10,11], and there are now gene therapy approaches for Superoxide Dismutase (SOD) 1 positive ALS [12,13]. The field continues to shift towards genetic medicine.

Many research groups around the world are currently working to develop improved treatment options and medications. Many high-level practice-changing studies with implications in clinical care have been published in the past two years. Phase 3 of the EMBARK (Evaluation of Micro-dystrophin (delandistrogene moxeparvovec) in Boys with Ambulatory Recognized Knowledge of Duchenne Muscular Dystrophy) trial on adeno-associated virus-based gene therapy in DMD reported primary outcomes in 2024, informing eligibility and expectations for non-ambulatory patients [14]. Tofersen for SOD1-ALS showed sustained effects in real-world 12-month data from the German early access program, expanding clinical use beyond trial populations [15]. Givinostat, a histone deacetylase inhibitor, demonstrated safety and efficacy in boys with DMD in phase 3 of the EPIDYS (Epigenetic Rescue of Dystrophin Dysfunction) trial [16], leading to regulatory approval in the United States in 2024. Vamorolone provided 48-week efficacy and safety data as a corticosteroid alternative for treating DMD [17].

These ongoing scientific advances will most likely lead to many patients having better prognosis and longer life expectancy in the future.

It is important to focus intensively on providing the best possible clinical care for these patients in addition to scientific efforts. A Swiss participatory mixed-methods study (2020–2025) developed and implemented an evidence-based, family-centered care management model for neuromuscular disorders across eight centers. Expected outcomes include improved care coordination, quality of life, self-efficacy, and reduced caregiver burden [18].

It is also important to pay more attention to the rehabilitative needs of affected individuals, especially since there is little evidence-based knowledge in this area. However, an increasing number of high-quality studies are being published in prestigious journals in this field. A randomized study conducted in 2025 showed that personalized home-based aerobic exercise with coaching is safe and effective for adults with various neuromuscular disorders, including muscular dystrophies, post-polio syndrome, and Charcot–Marie–Tooth disease. A total of 91 participants over 18 months showed improved fitness and integrated activity into daily life [19]. It will also become increasingly important for rehabilitation physicians to consider acute medical aspects and modern molecular genetic therapies. The enormous economic significance and challenges of these very expensive therapies will be briefly mentioned here but not discussed.

Given these developments, it is very clear that neuromuscular disorders are by no means neglected nowadays. It is a rapidly evolving field; while therapeutic options are increasing, a great deal of work and research must be conducted.

For this reason, this innovative Special Issue focuses on current scientific aspects of modern clinical care and rehabilitation for neuromuscular diseases. This topic is highly relevant!

## 3. Insights from This Special Issue

In this Special Issue, renowned experts present their work on topics related to clinical care and rehabilitation for neuromuscular diseases.

Aksel-Kilicarslan and colleagues [20] present the first combined histological and proteomic analysis of the quadriceps muscle in a patient carrying a pathogenic KMT5B variant with a neuromuscular phenotype. Pathogenic variants in KMT5B, a histone lysine methyltransferase, have been linked to neurodevelopmental disorders, but their effects on human skeletal muscle remained unexplored. Their findings contribute to a deeper understanding of the pathological changes in the affected muscles, which could positively impact the development of therapeutic approaches in the future.

Vianello et al. focus on a very practical aspect of the clinical care of patients with spinal muscular atrophy, analyzing the effects of long-term non-invasive ventilation on tracheostomy-free survival and hospitalizations [21]. Di Martino and colleagues focus on complement inhibitors, a modern group of drugs used to treat myasthenia gravis [22]. This Special Issue also presents papers focusing on modern diagnostic methods and evidence-based treatments in rehabilitation. The included articles cover a wide range of topics, providing valuable insights into pathophysiology, diagnosis, and treatment. Some articles address complex diseases in general, such as rehabilitation in ALS, while others focus on specific aspects. This diversity reflects the broad spectrum and multitude of possible and important questions surrounding neuromuscular diseases. All contributions greatly expand our understanding of this group of diseases.

## 4. What Challenges and Research Priorities Lie Ahead?

Due to the large number of diseases and the constant discovery of new disease entities, there is still much work to be done in this area, both clinically and scientifically. Gene therapy approaches are still far from reaching their goal; thus, ongoing work is needed in this area. Unsolved problems in neuromuscular disorder care include a lack of disease-modifying therapies for most rare conditions, inadequate multidisciplinary care coordination, and the need for optimized, tailored rehabilitation protocols.

Key challenges also include managing respiratory/swallowing dysfunction, mitigating psychosocial morbidity, improving access to specialized care, and utilizing telemedicine.

The main socioeconomic challenge will be making innovative therapies affordable for patients and healthcare systems around the world, as some options, particularly gene therapy approaches, are associated with exorbitantly high costs which has sparked global controversy [23].

However, scientific work in the field of neuromuscular diseases is highly rewarding for neuroscientists due to the wide range of possibilities, and ultimately, all advances benefit our patients. The advances of recent decades offer hope that many more innovations will be realized in the future.

## Abbreviations

ALS	Amyotrophic Lateral Sclerosis
DMD	Duchenne Muscular Dystrophy
SOD	Superoxide Dismutase
